# Inhibition of SREBP1 sensitizes cells to death ligands

**DOI:** 10.18632/oncotarget.239

**Published:** 2011-03-10

**Authors:** Yanina Eberhard, Marcela Gronda, Rose Hurren, Alessandro Datti, Neil MacLean, Troy Ketela, Jason Moffat, Jeffrey L. Wrana, Aaron D. Schimmer

**Affiliations:** ^1^ Princess Margaret Hospital, Ontario Cancer Institute, Toronto, ON, Canada; ^2^ Mount Sinai Hospital, Toronto, ON, Canada; ^3^ Department of Molecular Genetics, Donnelly Centre for Cellular and Biomolecular Research, Toronto, ON, Canada

**Keywords:** death receptor pathway of caspase activation, FASL, TRAIL, SREBP1, Orlistat

## Abstract

Evasion of death receptor ligand-induced apoptosis contributs to cancer development and progression. To better understand mechanisms conferring resistance to death ligands, we screened an siRNA library to identify sequences that sensitize resistant cells to fas activating antibody (CH-11). From this screen, we identified the Sterol-Regulatory Element-Binding Protein 1 (SREBP1), a transcription factor, which regulates genes involved in cholesterol and fatty acid synthesis including fatty acid synthase. Inhibition of SREBP1 sensitized PPC-1 and HeLa to the death receptor ligands CH-11 and TRAIL. In contrast, DU145 prostate cancer cells that are resistant to death ligands despite expressing the receptors on their cell surface remained resistant to CH-11 and TRAIL after knockdown of SREBP1. Consistent with the effects on cell viability, the addition of CH-11 activated caspases 3 and 8 in HeLa but not DU145 cells with silenced SREBP1. We demonstrated that knockdown of SREBP1 produced a marked decrease in fatty acid synthase expression. Furthermore, genetic or chemical inhibition of fatty acid synthase with shRNA or orlistat, respectively, recapitulated the effects of SREBP1 inhibition and sensitized HeLa but not DU145 cells to CH-11 and TRAIL. Sensitization to death receptor ligands by inhibition of fatty acid synthase was associated with activation of caspase 8 prior to caspase 9. Neither silencing of SREBP1 or fatty acid synthase changed basal expression of the core death receptor components Fas, caspase 8, FADD, caspase 3 or FLIP. Thus, inhibition of SREBP1 or its downstream target fatty acid synthase sensitizes resistant cells to death ligands.

## INTRODUCTION

Effector caspases can be activated through several mechanisms including the death receptor pathway. In this pathway, death receptor ligands such as Fas ligand (FasL) and TNF-related apoptosis-inducing ligand (TRAIL) bind cell surface receptors leading to the dimerization and activation of the upstream caspase, caspase-8 with the aid of the adapter protein, FADD. Activated caspase-8 then cleaves and activates caspase-3 with or without amplification through the mitochondrial pathway of caspase activation [1-3]. Defects in this signaling pathway can render cells resistant to death receptor ligands and render malignant cells resistant to TRAIL, thereby limiting the clinical efficacy of this experimental therapeutic agent.

Previous studies by our group and others have demonstrated that over-expression of the caspase-8 inhibitor FLIP overcomes resistance to death receptor ligands [4-8] and chemical or genetic inhibition of FLIP restores sensitivity to death ligands in some cell lines [8-12]. In other *in vitro* models, resistance to death receptor ligands has been attributed to over-expression of FAP-1, the protein-tyrosine phosphatase which interacts with Fas and prevents Fas translocation to the cell surface [13-14]. Alternatively, resistance to death ligands has been also linked to somatic mutations in caspase 8 [15-17].

To identify additional strategies to overcome resistance to death receptor stimuli, we screened an siRNA library to identify sequences that sensitize resistant cells to CH-11. From this screen, we identified the Sterol-Regulatory Element-Binding Protein1, SREBP1. This gene encodes a transcription factor that binds to the sterol regulatory element-1 (SRE1), thereby regulating multiple genes involved in fatty acid and sterol biosynthesis including fatty acid synthase and HMGCoA reductase [18-19]. Here, we demonstrated that silencing of SREBP1 restored sensitivity to CH-11 and TRAIL through a mechanism at least partly related to inhibition of fatty acid synthase expression. Thus, this study highlights novel mechanisms to overcome resistance to death receptor ligands.

## RESULTS

### Identification of siRNA that sensitize resistant cells to CH-11

To identify genetic targets whose inhibition restores sensitivity to death receptor ligands, a cell-based high throughput screen was performed using the FasL and TRAIL-resistant prostate cancer cell line PPC-1 and the commercially available Dharmacon siRNA library of 6080 SMARTpools. Screens were performed in 96 well plates to which siRNA were added at 40nM followed 6 hours later by the addition of agonistic anti-Fas monoclonal antibody (CH-11) (50 ng/mL). Cell viability was measured 24 hours after siRNA transfection by MTS assay. Each plate included controls of untreated cells, cells treated only with CH-11, and cells transfected with siRNA control. From this screen, we identified 64 genes (1%) that decreased viability at least 3 standard deviation away from the mean B score of the entire population of tested siRNA. These 64 siRNA were retested in secondary assays. Twenty of the 64 hits were reproducible on repeat testing and induced cell death in the presence of CH-11. These 20 siRNA sequences were retested in the presence and absence of CH-11 to identify FasL sensitizers. Of these 20 siRNA sequences, 2 sequences reduced cell viability in the presence of CH-11 > 50% compared to cells treated with control buffer. The other 18 had lesser degrees of sensitization. Of these 2 sequences, one was FLIP (65% reduction in viability in the presence of CH-11) and the other was SREBP1 (57% reduction in viability in the presence of CH-11). Previously, we demonstrated that chemical or genetic knockdown of FLIP sensitizes resistant cells to CH-11 [8], thus, validating the efficacy of our siRNA screen. Therefore, we investigated SREBP1 as a potential FasL sensitizer.

### Silencing of SREBP1 sensitizes resistant tumor cells to death receptor ligands

**Figure 1:**
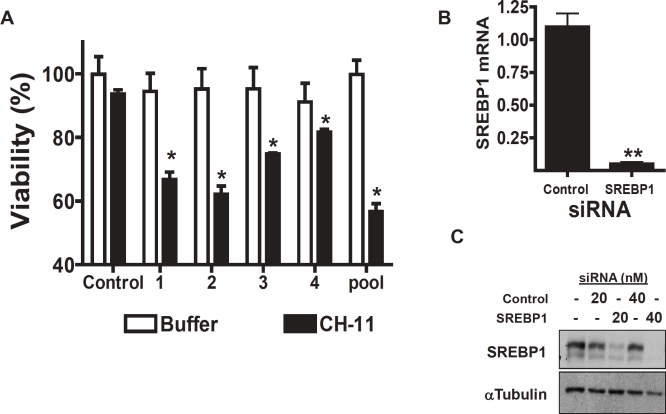
Genetic silencing of SREBP1 sensitizes resistant cells to CH-11 **A) PPC-1 cells were transfected with four individual siRNA duplexes targeting SREBP1, the Dharmacon SMARTpool or control sequences (final concentration 40nM).** Six hours after transfection, cells were treated with CH-11 (50 ng/mL) or buffer control for 18 hours. After incubation, cell growth and viability was measured with the MTS assay. Data represent the mean + SD of a representative experiment performed in triplicate (* p<0.05, Student t test). **B) SREBP1 mRNA expression was measured relative to 18S RNA by real-time RT-PCR.** Data points represent the mean ± SD percent of SREBP1/18S expression relative to controls (△△CT normalization) from independent experiments performed in triplicate. (** p<0.01, Student t test). **C) PPC-1 cells were transfected with SREBP1 SMARTpool siRNA or control sequences.** 24 hours after transfection, total cellular proteins were isolated and analyzed by SDS-PAGE immunoblotting using antibodies against SREBP1 and tubulin.

Having identified SREBP1 in our siRNA screen, we tested the ability of four individual siRNA duplexes targeting SREBP1 to sensitize cells to CH-11. All 4 of the individual duplexes as well as the pooled siRNA sensitized the resistant PPC-1 cells to CH-11 and decreased expression of SREBP1 protein and mRNA. In contrast, no sensitization to CH-11 or knockdown of SRERP1 was observed after transfection of control siRNA. (Figure 1). Of note, SREBP1 knockdown did not sensitize PPC-1 cells to VP-16, a stimulus of the mitochondrial pathway of caspase activation (data not shown).

**Figure 2:**
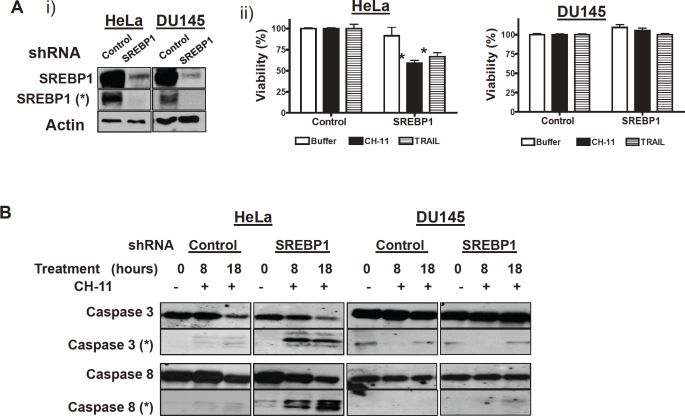
Genetic inhibition of SREBP1 sensitizes cells to CH-11 and TRAIL via caspase activation **A) HeLa and DU145 were infected with shRNA targeting SREBP1 or control sequences and stable populations selected**; **i) Total cellular proteins were isolated and analyzed by SDS-PAGE immunoblotting using antibodies against SREBP1 and actin.** * represents the cleaved form of SREBP1 (68 Kda). **ii) Cells stably infected with shRNA targeting SREBP1 or control sequences were treated with CH-11 (100 ng/mL for 18 hours) or TRAIL (100 ng/mL 3 hours).** After incubation, cell growth and viability was measured by the MTS assay. Data represent the mean + SD of a representative experiment performed in triplicate (* p<0.05, Student t test). **B) The same cells infected as above were treated with CH-11 (100 ng/mL).** At increasing times after incubation, total cellular proteins were isolated and analyzed by SDS-PAGE immunoblotting using antibodies against caspase-3, caspase-8. * represents cleaved caspase 3 (17-22Kda) and cleaved caspase 8 (36/40 Kda).

To validate the effects of SREBP1 knockdown on death receptor sensitization and determine the effects of its knockdown in other cell lines, HeLa cervical cancer and DU145 prostate cancer cells were stably infected with shRNA targeting SREBP1 or control sequences. Previously we and others have demonstrated DU145 and HeLa cells are resistant to death ligands despite expressing death receptors on the cell surface [8, 20-21]. HeLa but not DU145 cells are sensitized to death ligands by knockdown of the caspase-8 inhibitor FLIP [8, 20-21]. SREBP1 target knockdown in both cell lines was confirmed by immunoblotting. Moreover, immunoblotting demonstrated knockdown of both the precursor and active cleaved forms of SREBP1 (Figure 2Ai). To evaluate the effects of SREBP1 inhibition on CH-11 and TRAIL sensitization, HeLa and DU145 cells stably expressing SREBP1 shRNA or control sequences were treated with CH-11, TRAIL or buffer control. After treatment, cell growth and viability was measured by the MTS assay. In HeLa but not DU145 cells, knockdown of SREBP1 sensitized cells to both CH-11 and TRAIL (Figure 2Aii). Reductions in cell viability were confirmed by PI staining and flow cytometry.

Given the ability of SREBP1 knockdown to sensitize cells to death ligands, we examined the effects of target knockdown on caspase activation. Consistent with the effects on cell viability, we observed cleavage of caspases 3 and 8 and reductions in the pro-forms of these caspases in HeLa cells with silenced SREBP1 treated with CH-11. In contrast, no changes in cleaved caspases 3 and 8 or the pro-forms were observed in DU145 cells that were resistant to CH-11 sensitization by SREBP1 inhibition. Of note, no significant changes in these caspases were noted in cells treated with CH-11 alone or with knockdown of SREBP1 alone (Figure 2B).

### Genetic and chemical inhibition of fatty acid synthase sensitizes resistant cells to death receptor ligands

**Figure 3:**
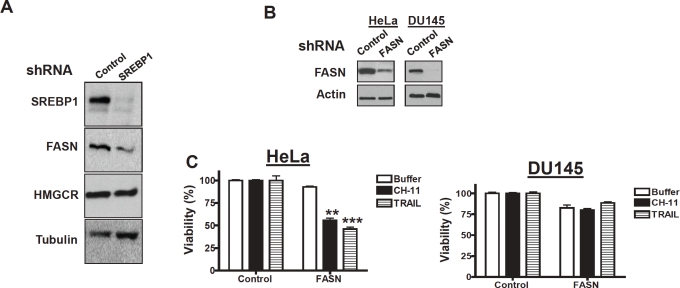
Genetic knockdown of FASN sensitizes cells to death ligands **A) HeLa and DU145 cells were infected with shRNA targeting SREBP1 or control sequences and stable populations selected as described in materials and methods Total cellular proteins were isolated and analyzed by SDS-PAGE immunoblotting using antibodies against SREBP1, fatty acid synthase (FASN), 3-hydroxy-3-methylglutaryl-Coenzyme A reductase (HMGCR) and tubulin**. **B) HeLa and DU145 cells were infected with shRNA targeting FASN or control sequences and stable populations selected.** Total cellular proteins were isolated and analyzed by SDS-PAGE immunoblotting using antibodies against FASN and actin. **C) HeLa and DU145 cells infected with FASN or control shRNA as in (B) were treated with CH-11 (100 ng/mL for 18 hours), TRAIL (100ng/mL for 3 hours), or buffer control.** After incubation, cell growth and viability was measured by the MTS assay.

SREBP1 positively regulates the expression of genes involved in cholesterol and fatty acid synthesis including fatty acid synthase (FASN) and HMG CoA reductase (HMGCR)[22-23]. Therefore, we examined changes in FASN and HMG CoA protein expression in HeLa cells with stable knockdown of SREBP1. In HeLa cells with stable knockdown of SERBP1 we observed a marked decrease in FASN expression compared to control cells. However, no change in HMGCR expression was detected (Figure 3A).

**Figure 4:**
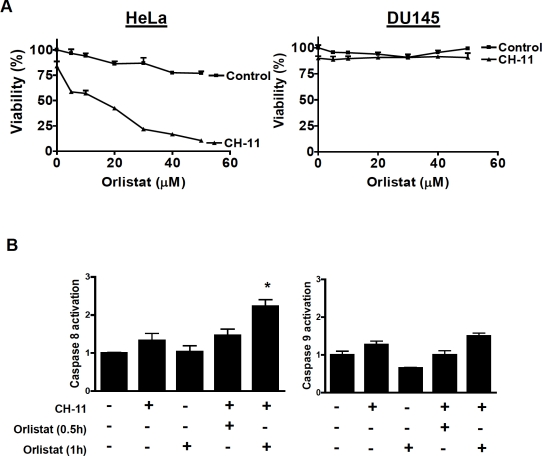
Chemical inhibition of FASN sensitizes to CH-11 and activates caspases **A) HeLa and DU145 cells were treated with increasing concentrations of Orlistat and CH-11 (100 ng/mL) for 18 hours.** After incubation cell growth and viability was measured by MTS assay. Data represent the mean + SD of a representative experiment performed in triplicate. **B) HeLa cells were incubated with CH-11 (100 ng/mL final concentration) and/or Orlistat (50 μM final concentration) for 0.5 and 1 hour.** After incubation, activation of caspases 8 and 9 were assessed by Flow cytometry as described in materials and methods. * p<0.05, Student t test.

Therefore, we tested whether silencing of FASN could recapitulate the effects of SREBP1 knockdown and sensitize cells to death receptor ligands. HeLa and DU145 cells were infected with shRNA targeting FASN or control shRNA and stable populations of cells selected. Knockdown of FASN protein was confirmed by immunoblotting (Figure 3B). Knockdown of FASN sensitized HeLa to CH-11 and TRAIL, but DU145 cells remained resistant (Figure 3C). As a chemical approach to confirm the ability of FASN inhibition to sensitize cells to death ligands, HeLa and DU145 cells were treated with increasing concentrations of the chemical FASN inhibitor, orlistat (S)-2-formylamino-4-methyl-pentanoic acid (S)-1-[[(2S, 3S)-3-hexyl-4-oxo-2-oxetanyl] methyl]-dodecyl ester [24], with and without CH-11 or TRAIL. Orlistat sensitized HeLa cells to CH-11 and TRAIL but had no effect on DU145 cells (Figure 4A). We also measured caspase activation in HeLa cells treated with orlistat and CH-11. Caspase-8 activation was observed within 1 hour of treating HeLa cells with orlistat and CH-11 and activation of caspase-8 preceded activation of caspase-9. Of note no significant increase in caspase activation was seen in HeLa cells treated with either orlistat or CH-11 alone (Figure 4B). Thus, chemical inhibition of FASN mimics the observations with genetic silencing of FASN and SREBP1.

**Figure 5:**
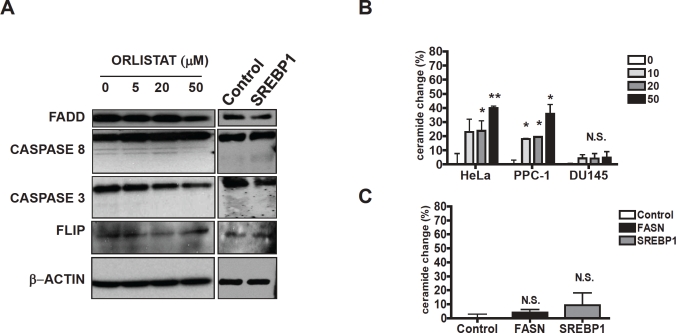
Effects of SREBP1 or FASN inhibition on levels of core components of the death receptor pathway of caspase activation and ceramide **A) FADD, Caspase 8, Caspase 3 and Flip were studied in HeLa cells treated with increasing concentrations of Orlistat or infected with SREBP1 or Control shRNA.** Actin was used as loading control. **B) Membrane ceramide expression was analized by Flow Cytometry after increasing concentrations of Orlistat for 24 hours or after FASN, SREBP1 or Control silencing shRNA in HeLa cells.** *p<0.05, Student t test.

To understand the mechanism by which inhibition of SREBP1 and FASN sensitize cells to death ligands, we measured changes in cell surface expression of the Fas in HeLa cells with knockdown of SREBP1 or treated with orlistat. Inhibition of SREBP1 expression with shRNA and inhibition of FASN with orlistat did not significantly change the abundance of Fas on the plasma membrane by flow cytometry (0.9 + 0.05 fold change in geometric mean of Fas expression after 50μM Orlistat and 1.23 + 0.18 fold change geometric mean after SREBP1 silencing, both p>0.05 by Student t test). Likewise, we could not detect changes in Fas localization after SREBP1 or FASN knockdown by shRNA as examined by confocal microscopy (data not shown). We also measured expression of FADD, FLIP, caspase-8 and caspase-3 expression in HeLa cells with knockdown of SREBP1 or treated with orlistat. Again, no differences in expression were detected (Figure 5A). Thus, inhibition of SREBP1 and FASN does not sensitize cells to death ligands by changing expression of core components of the death receptor pathway.

Inhibition of FASN with orlistat has been associated with increased production of ceramide [25]. Therefore, we measured changes in ceramide after inhibition of FASN and SREBP1. Treatment of HeLa cells with orlistat increased production of membrane ceramide at concentrations associated with sensitization to death ligands. However, genetic knockdown of FASN or SREBP1 did not increase ceramide production (Figure 5B). In addition, the ceramide inhibitors Desipramine [26], GW4869 [27] and Fumoisin B1 [28] did not abrogate the ability of orlistat to sensitize HeLa cells to CH-11 and TRAIL, and supplementation with external ceramide did not sensitize the cells to CH-11 (data not shown). Thus, in this model, increased ceramide production does not appear sufficient to explain sensitization to death receptor ligands after SREBP1 and FASN inhibition.

## DISCUSSION

Defects in the death receptor pathway of caspase activation can render cells resistant to death ligands and thereby limit the clinical efficacy of agents such as TRAIL. To better understand mechanisms of resistance to death ligands and define strategies to overcome such blocks, we conducted an siRNA screen to identify sequences that sensitize resistant cells to CH-11. From this screen we identified SREBP-1. SREBP1 is a transcription factor that, upon cleavage to an active form, regulates genes involved in lipid and cholesterol metabolism [22, 29]. Consistent with its role in lipid metabolism, transgenic mice over-expressing active SREBP-1 developed fatty livers with increased triglycerides and cholesteryl esters [30]. In chromatin precipitation experiments, SREBP1 interacts with target genes related to lipid and insulin metabolism. These experiments also demonstrated that SREBP1 interacts with the cell cycle regulators CDK2 and E2F4 [31]. While the functional significance of this interaction with cell cycle regulators was unclear, these results suggest that SREBP1 may also regulate pathways beyond lipid metabolism.

Over-expression of SREBP1 appears related to the pathogenesis and progression of some malignancies [32-35]. For example, up regulation of SREBP1 and its target genes has been associated with progression to androgen independence in prostate cancer [36]. In addition, gene expression studies have demonstrated up-regulation of SREBP1 and its target genes in the putative stem cell fraction of prostate cancer [37]. It is not fully understood, however, how the dysregulation of lipid and cholesterol pathways promotes malignant transformation. Nonetheless, these data further support targeting these pathways as a therapeutic approach for the treatment of malignancy.

SREBP1 is related to a homologous transcription factor SREBP2, but the genes under their control differ. For example, SREBP1 mainly regulates genes associated with fatty acid synthesis and phospholipid pathways while SREBP2 primarily regulates genes associated with cholesterol synthesis and uptake [38-39]. Of note, in our study, we did not identify SREBP2 as a hit in our siRNA screen.

Previously, we and others have demonstrated that over-expression of the endogenous dominant negative homolog of caspase-8, FLIP (c-Fas-associated death domain-like IL-1-converting enzyme-like inhibitory protein) is one mechanism by which malignant cells become resistant to stimuli of the death receptor pathway of caspase activation [40-43]. We also demonstrated that inhibition of FLIP protein or mRNA synthesis is sufficient to sensitize some cells to the death receptor ligands CH-11 and TRAIL [8]. Therefore, we examined the effects of down-regulating SREBP1 on the expression of FLIP. No change in FLIP expression was detected, indicating that inhibition of SREBP1 sensitizes cells to CH-11 and TRAIL independent of changes in FLIP. Rather, sensitization to death ligands appears related, at least in part, to reductions in fatty acid synthase expression. In support of this contention, SREBP1 reduced expression of FASN and genetic or chemical inhibition of FASN recapitulated the effects of SREBP1 knockdown.

Fatty acid synthase (FASN) catalyzes the synthesis of palmitate from acetyl CoA, malonyl CoA and NADPH [44]. In normal tissues, except for cycling endometrium and lactating breast tissue, levels of FASN are low to undetectable [45-46], as the source of fatty acids is primarily the diet [46]. In contrast, malignant cells have high levels of FASN [47-50], potentially reflecting the higher metabolic demands of these tumors. In some malignant cells, chemical or genetic silencing of FASN induces cell death *in vitro* and delays tumor growth *in vivo* [51-52]. Some of these studies report that blocking FASN induces apoptosis through a mechanism linked to increased ceramide production [25]. Therefore, we examined ceramide production in our cells system. While we also demonstrated increases in ceramide after orlistat treatment, this increase did not appear functionally important for sensitization to death ligands. In support of this contention, genetic knockout of SREBP1 or FASN did not increase levels of ceramide. In addition, inhibitors of ceramide production did not protect against orlistat-mediated sensitization to CH-11. Finally, supplementation of the medium with ceramide did not sensitize cells to CH-11. This observation is in agreement with a study by Knowles et al., who also demonstrated that cell death induced by orlistat was associated with activation of caspase-8 but independent of ceramide[53].

In our study, inhibition of SREBP1 or FASN did not sensitize DU145 cells to CH-11 or TRAIL despite the presence of the death receptors on the cell surface and target knockdown by RNAi. Yet, we demonstrated target knockdown in these resistant cells. Therefore, further work will be required to understand the basis for resistance in these lines how SREBP1 and FASN knockdown sensitize other cell lines to death receptor ligands. One possibility is that SREBP1 and FASN knockdown alterns the organization of the plasma membrane and lipids rafts, thus changing the localization of the death receptors associated to the membrane [54]. Although we could not show changes in Fas localization by confocal microscopy, more detailed studies on Fas localization using isolated lipid rafts might demonstrate changes in death receptor localization.

The findings of this study could have therapeutic implications. Potentially, the clinical efficacy of TRAIL as a treatment for malignancy could be improved by combining TRAIL with SREBP1 or FASN inhibitors. While small molecule inhibitors of SREBP1 are not available, orlistat is an FDA-approved chemical inhibitor of FASN and could be combined with TRAIL. However, in its current formulation, orlistat is poorly absorbed after oral administration [55] therefore an oral formulation with improved bioavailability or an intravenous formulation of orlistat would be required for the treatment of maligancy. Thus, in summary, this work highlights mechanisms of resistance to death ligands and strategies to overcome this resistance.

## MATERIALS AND METHODS

### Cell lines

PPC-1 and DU145 prostate cancer cell lines and HeLa cervical cancer cells were cultured in RPMI 1640 medium. All cells were supplemented with 10% fetal bovine serum (FBS) (Hyclone, Logan, UT), penicillin (500 IU/mL), and streptomycin (50 μg/mL). All cells were cultured at 37 °C in a humid atmosphere with 5% CO_2_.

### Reagents

The monoclonal antibody anti-Fas (CH-11) was purchased from MBL International Corporation (Woburn, MA) and soluble killer TRAIL was purchased from Alexis Biochemicals (San Diego, CA). Orlistat, GW4869, Fumonisin B1 and Desipramine hydrochloride were purchased from Sigma (St. Lois, MO). C16 ceramide was obtained from TRC (North York, ON) and was dissolved in isopropanol.

### Viability assays

The CellTiter96 aqueous nonradioactive MTS assay was used to measure cell growth and viability according to the manufacturer's instructions (Promega) and as described previously [56]. Propidium iodide (PI) staining was performed according to manufacturer's instructions (Biovision, Mountain view, CA).

### siRNA screen

PPC-1 cells were plated in 96-well plates in complete RPMI medium 18 hours before the siRNA transfection. After adhering to the plates, medium was removed and cells were treated with aliquots of the the Human Druggable siRNA library (6080 siRNA pools) (Dharmacon, IL) (final concentration 40 nM) with lipofectamine 2000 (Invitrogen, CA) in medium without FBS or antibiotics. After 6 hours of incubation, CH-11 was added (50ng/mL final concentration) along with FBS and media. Eighteen hours later and 24 hours after siRNA transfection, cell viability was measured by the MTS assay. Results were normalized and corrected for systematic errors using the B score[57]. Compounds with a B score value lower than 3 times the standard deviation were empirically considered hits in the assay.

Plate handling was performed by a CRS Dimension4 robotics platform equipped with a Linear Plate Transport system (LPT) (Thermo Electron, MA). Plate transfer from the LPT to online peripherals was carried out by a CRS Flip Mover and Vertical Array Loader (Thermo Electron). Liquid handling steps were performed by a Biomek FX Laboratory Automation Workstation (Beckman Coulter) and ELx405 Magna cell washers (Biotek, Vermont). Robotics integration was controlled by a Polara integration software (Thermo Electron).

### SREBP1 siRNA transfection

Cells were transfected with siRNA targeting SREBP1 as above using the SREBP1 SMARTpool (Dharmacon) and individual sets of siRNA (Dharmacon):

Set 1
  sense, 5'-UGACUUCCCUGGCCUAUUUUU-3', antisense, 5'-AAAUAGGCCAGGGAAGU CAUU-3';
Set 2
  sense, 5'-ACAUUGAGCUCCUCUCUUGUU-3', antisense, 5'-CAAGAGAGGAGCUCAAUGUUU-3',
Set 3
  sense, 5'-GCGCACUGCUGUCCA CAAAUU-3', antisense, 5'-UUUGUGGACAGCAGUGCGCUU-3';
Set 4
  sense, 5'-ACACAGACGUGCUCAUGGAUU-3', antisense, 5'-UCCAUGAGCACGUCU GUGUUU-3'.

Cells transfected with control siRNA (Control #2, Dharmacon) was used during the experiments.

### shRNA infection

Construction of hairpin-pLKO.1 vectors (carrying a puromycin antibiotic resistance gene) containing shRNA sequences and production of short hairpin RNA viruses has been described in detail [58]. The shRNA targeting the SREBP1, FASN and control coding sequences are as follows: SREBP1, 5'-GCCATCGACTACATTCGCTTT-3'; FASN, 5-CATGGAGCGTATCTGTGAGAA-3' and Control 5'-TGCCCGACAACCACTACCTGA-3'. Lentiviral infections were performed essentially as described [59]. Briefly, adherent cells were treated with 0.5 mL of the virus, followed by overnight incubation (37 °C, 5% CO_2_) without removing the virus. The next day, viral media was replaced with fresh media containing puromycin (2 μg/mL) to select a population of resistant cells.

### Quantitative real-time polymerase chain reaction

First-strand cDNA was synthesized from 1 μg of DNase-treated total cellular RNA using random primers and SuperScript II reverse transcriptase (Invitrogen) according to the manufacturer's protocols. Real-time PCR assays were performed in triplicate with 5 ng of RNA equivalent cDNA, SYBR Green PCR Master mix (Applied Biosystems, Foster City, CA, USA), and 400 nM of gene-specific primers. Reactions were processed and analyzed on an ABI 7900 Sequence Detection System (Applied Biosystems). Forward/reverse PCR primer pairs for human cDNAs were as follows:: SREBP1, forward, 5'-GCAAGGCCATCGACTACATT-3'; reverse, 5'-GGTCAGTGTGTCCTCCACCT; FASN, forward, 5'-CTGGCTCAGCACCTCTATCC-3', reverse, 5'-CTCCAGGTTGTCCCTGTGAT-3'; and 18S, forward, 5'-AGGAATTGACGGAAGGGCAC-3'; reverse, 5'-GGACATCTAAGGGCATCACA -3'. Relative mRNA expression was determined using the △△CT method as described [60].

### Immunoblotting

Total cell lysates were prepared from cells as described previously [60]. Briefly, cells were washed with phosphate-buffered saline pH 7.4, and suspended in lysis buffer (10 mmol/L Tris, pH 7.4, 150 mmol/L, NaCl, 0.1% Triton X-100, 0.5% sodium deoxycholate, and 5 mmol/L EDTA) containing protease inhibitors (Complete tablets; Roche, IN). Protein concentrations were measured by the Bradford assay [61]. Equal amounts of protein were subjected to sodium dodecyl sulphate polyacrylamide gel electrophoresis (SDS page) followed by transfer to polyvinylidene difluoride (PVDF) or nitrocellulose membranes. Membranes were probed with rabbit anti—SREBP1 (2A4) (1:1000 [v/v]; Santa Cruz Biotechnologies, CA), mouse anti—FASN (1:1000 [v/v]), anti-FADD (1:2000 [v/v]), anti-caspase 8 (1:1000 [v/v]) (BD Transduction labs, San Jose, CA), rabbit anti-caspase 3 (Cell Signaling, Danvers, MA) β-actin (1:20000 [v/v]) or α-tubulin (1:10000 [v/v]) (Sigma, St. Lois, MO), anti-FLIP (NF6) (1:500 [v/v]) ((Alexis Biochemicals, San Diego, CA) or anti-HMGCR (clone A9) (1:1000 [v/v]) (kindly provided by Dr LZ Penn, Ontario Cancer Institute, Toronto, Canada). Secondary antibodies were horseradish peroxidase-conjugated goat anti mouse IgG (1:10 000 [v/v]) and anti rabbit (1:5000 [v/v]) (GE Healthcare, Chalfont St Giles, United Kingdom). Detection was performed by the enhanced chemical luminescence method (Pierce, Rockford, IL).

### Fas and ceramide expression

Fas expression was measured using mouse anti-human Fas FITC and mouse IgG1 FITC (BD and Co., Mountain View, CA). Ceramide expression was measured with mouse anti-human ceramide (MID 15B4) (Alexis Biochemicals, San Diego, CA), followed by FITC rat anti-mouse Ig/M (BD and Co., Mountain View, CA). Cells were analyzed with a Becton Dickinson FACSCalibur (BD and Co., Mountain View, CA) flow cytometer and analyzed by FlowJo analysis software (Tree Star, Ashland, OR).

### Caspase activation assay

Activation of caspases 8 and 9 was detected by APO LOGIX carboxyfluorescein caspase detection kits (Cell Technology, Mountain View, CA) following the manufacturers instructions [62].
